# Influence of the Time Scale on the Construction of Financial Networks

**DOI:** 10.1371/journal.pone.0012884

**Published:** 2010-09-30

**Authors:** Frank Emmert-Streib, Matthias Dehmer

**Affiliations:** 1 Computational Biology and Machine Learning, Center for Cancer Research and Cell Biology, School of Medicine, Dentistry and Biomedical Sciences, Queen's University Belfast, Belfast, United Kingdom; 2 Institute for Bioinformatics and Translational Research, The Health and Life Sciences University (UMIT), Hall in Tyrol, Austria; University of East Piedmont, Italy

## Abstract

**Background:**

In this paper we investigate the definition and formation of financial networks. Specifically, we study the influence of the time scale on their construction.

**Methodology/Principal Findings:**

For our analysis we use correlation-based networks obtained from the daily closing prices of stock market data. More precisely, we use the 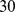 stocks that currently comprise the Dow Jones Industrial Average (DJIA) and estimate financial networks where nodes correspond to stocks and edges correspond to none vanishing correlation coefficients. That means only if a correlation coefficient is statistically significant different from zero, we include an edge in the network. This construction procedure results in unweighted, undirected networks. By separating the time series of stock prices in non-overlapping intervals, we obtain one network per interval. The length of these intervals corresponds to the time scale of the data, whose influence on the construction of the networks will be studied in this paper.

**Conclusions/Significance:**

Numerical analysis of four different measures in dependence on the time scale for the construction of networks allows us to gain insights about the intrinsic time scale of the stock market with respect to a meaningful graph-theoretical analysis.

## Introduction

Financial markets are one of the most fascinating and complex systems of our times. Investigating such markets is important not only because a more and more globalizing world depends strongly on the cautious regulation of these markets allowing their proper functioning. But also because we might be able to gain insights that are fruitful and beneficial for our understanding of complex adaptive systems in general [1]–[3]. For this reason, during the last decades, questions in quantitative finance and econophysics have attracted many scientists from diverse fields, e.g., physics, computer science and statistics [4], [5] to study this exciting phenomenon.

One result of this effort, so far, is the understanding that the usage of networks and network-based concepts are beneficial in the study of financial markets because they can be used as appropriate representation thereof [6]–[9]. This result coincides with findings in many other fields, e.g., biology or sociology where it has been realized that collective phenomena spanning large parts of a system are best studied coherently by means of their network structure [10]–[14]. For the stock market, the definition of *financial networks* is very frequently based on the correlation matrix of the composing stocks or companies. Whereas nodes correspond to stocks and edges are obtained from the correlation coefficients, either from a filtering or a transformation mapping. Especially, trees have been studied numerously [8], [15], because the concept of *minimum spanning trees* provides a procedure for the extraction of a tree from the correlation matrix. Further studies based on financial networks investigated the hierarchical structure of the market, clustered its constituting companies, studied the topology of the obtained trees or networks, or investigated the time-dependence of the observed correlations [6], [8], [15]–[18]. It is important to note that the way a financial network is constructed from the correlation matrix is not unique. For example, Mantegna [8] suggested to extract the minimum spanning tree (MST) to find the most important connections among all stocks, which also reveal their hierarchical organization. In contrast, Boginski et al. [6] constructed their network by thresholding the correlations resulting in different threshold dependent networks, and Onnela et al. [18] studied growing networks by adding successively edges according to their rank, ordered from strong to weak correlations. It is clear that the obtained trees or networks contain different, but possibly overlapping, information of the underlying market.

In this paper we address the question how financial networks should be constructed from given time series data of the stock market. More precisely, we study the influence of the time scale - the length of the time series - used to construct the networks. This question arises, because we do not want to construct one financial network for the whole time series, but we want to dissect the time series in 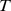 (non-overlapping) intervals of length 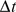 and construct a network for each interval. This is in contrast to, e.g., Mantegna [8] who constructed just one minimum spanning tree, but similar to Onnela [15] who used a sliding window resulting in overlapping intervals, for which minimum spanning trees have been extracted. In a none graph-theoretical context, Epps [19] was among the first who demonstrated that the correlations in the stock market - between stocks - decay if one goes to a time scale of hours or even shorter whereas the correlations increase for longer time scales. This is an important finding because this result suggests that the mixing of dependencies between stock prices depends strongly on the time scale to establish an interconnection within the market. It is not only important for our theoretical understanding of the stock market to learn about intrinsic time scales but also for practical applications, e.g., for time series prediction. The point is that in, e.g., multivariate time series analysis not only one, but multiple time series are used to predict the future outcomes of stock prices or their volatility. However, if there is no or only a very weak dependency between the utilized time series, the analysis could be reduced to the investigation of individual time series, because no cross information can be used to improve the prediction accuracy. Hence, significant correlations spanning the entire system are vital for such a multivariate analysis [20], [21]. The time scale of the stock market has been previously investigated by Kwapien et al. [22] who studied stocks from the NYSE and the Deutsche Börse. However, their focus was on the comparison of the evolution of contemporary with the historical market and their results demonstrate an acceleration in the relevant time scale. Also the mechanism that could explain the Epps-effect has been studied [23]. We want to emphasize, however, that none of these previous approaches is network-based.

The major contribution of this paper is to study the dependency of evolving financial networks on the time scale used for their construction. The financial networks we construct are correlation-based. However, instead of investigating properties of the correlation matrix directly, we study topological modifications of the extracted financial networks. These networks are undirected and unweighted, constructed statistically by a method recently introduced [7]. Our approach is motivated by results from systems biology where it has been demonstrated that the comparison of networks representing molecular pathways allows to study important modifications of functional units due to pathogenesis [24], [25]. Here the crucial point is that we hypothesize the topology of the financial networks is a reflection of the proper functional behavior of the stock market, rather than merely a mathematical auxiliary function. Certainly, this will depend crucially on the way the financial networks are constructed (defined). The goal of our analysis is to find a time scale that is most beneficial for such a definition of financial networks. That means our analysis can be seen as a preprocessing step for a further analysis of the obtained networks. The time scale used to estimate correlations is crucial, because for too low values the strength of the signal might be comparable to the noise in the data and, hence, the networks will not only be erroneous but also very sparse because of missing correlations. In the context of financial networks, this impression is suggested by the investigations of Epps [19]. On the other hand, if the time scale is too long, then we might reach saturation corresponding to an equilibrium. Hence, from this consideration it appears clear that we need to use intermediate values to construct (define) financial networks. The purpose of this article is to provide a quantitative investigation of the above argument.

This article is organized as follows. In the next section we introduce our methods and describe the financial data we use for our analysis. In the results section we present numerical results of our analysis. This article finishes with a discussion and conclusions.

## Methods

For our analysis we use data from the NYSE and the NASDAQ. More precisely, we use the daily closing prices from the stocks of the 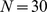 companies that currently comprise the Dow Jones Industrial Average (DJIA) for the time range starting at July 1986 and ending in December 2007 [26]. The starting date was chosen because Intel's stock was introduced in July 1986, as the last of the stocks considered in our analysis [7]. Because we want to study the evolution of financial networks we separate the time series in 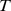 none overlapping intervals. Each interval 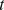 has a duration of 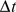 trading days. Table 1 shows the different parameters 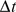 and 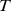 we study in this paper. The second column shows the number of consecutive days 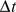 we use in our analysis to calculate the financial networks. The third column shows the number of resulting networks 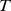, which equals the number of none overlapping intervals, from July 1986 to December 2007.

**Table 1:** The second column shows the number of consecutive days 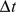 we used in our analysis to construct the networks.

C	number of days ( 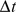 )	number of intervals ( 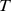 )
1.	5	996
2.	10	498
3.	20	249
4.	30	166
5.	40	124
6.	60	88
7.	100	53
8.	120	44
9.	240	22
10.	1000	5

The third column shows the number of resulting intervals respectively networks 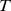 we obtain from July 1986 to December 2007. The first column is just a numbering of the studied cases (C).

In the following, we define how we construct a financial network for each interval 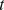. For simplicity, we omit the interval index 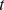 whenever possible. We start by transforming the time series of the prices 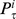 of stock 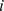 at day 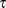 to log-return values [21] given by
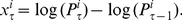
(1)From the obtained log-return values we calculate the Pearson product-moment correlation coefficient between pairwise stocks 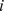 and 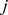 by
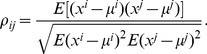
(2)The population correlation 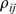 is estimated by the sample correlation [27]

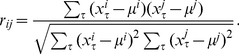
(3)In this paper, we are interested in the evolution of dependencies between pairs of stocks respectively their log-return values. We quantify this ‘dependency’ via Pearson's product-moment correlation coefficient of the log-return values. From the estimated correlation coefficients, see Eq. 3, we construct financial networks by applying undirectional statistical hypothesis tests to test for significance [28]. More precisely, a transformed value of the Pearson product-moment correlation coefficient follows a t-distribution with 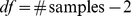 degrees of freedom. The hypothesis we are testing is
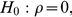
(4)against the alternative
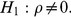
(5)From these tests we construct networks 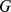 by setting [7]

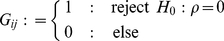
(6)A network 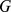 constructed this way is undirected and unweighted. That means, there will be an edge connecting node 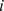 with node 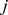 in 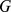, if the statistical test rejects the null hypothesis, given a significance level 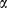, that the correlation coefficient 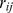 is zero [28]. In this paper we use the significance level 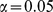. To our knowledge we are the first defining financial networks this way [7]. We want to stress that this definition allows a unique extraction of a network from the correlation matrix that is statistically significant and reproducible. Application of this procedure to all intervals gives 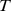 networks 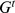, 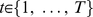. Each network 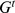 represents, thus, a certain time interval of trading activity and, hence, of the dynamics of the corresponding stock market. Certainly, an appropriate selection of 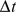 is crucial. For this reason, the influence of 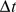 will be studied in the next section.

On a technical note, we want to remark that even for small sample sizes as well as non-normal data our experimental design allows for sound statistical estimations. In order to demonstrate this, we estimated the attained false positive rate of two uncorrelated random variables for two different cases. In the first case, the random variables are sampled from a normal distribution with mean 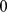 and a variance of 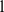, in the second case from a Pareto distribution with an exponent of 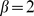. The Pareto distribution has a fat tail that decays accoring to a power law 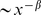 which may represent real data more appropriately. Averaging over 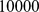 hypothesis tests, each for a sample size of 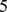 (because this is the smallest sample size used in our analysis, see table 1) and a significance level of 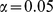 testing for a vanishing Pearson correlation coefficient gives for the normal data an estimated false positive rate of 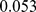 and for the Pareto distributed data 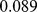. It is expected that the Pareto distributed data give worse results than the normal data, however, the attained false positive rate is also in this case acceptable [28].

## Results

In this section we present numerical results. We investigate three graph and one information-theoretical measure in order to analyze the influence of 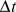 on the construction of the financial networks and their properties. The first measure quantifies the strength of the correlations on a system-wide scale - that means comprising all stocks - by calculating the *edge density* of the networks,
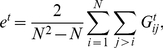
(7)

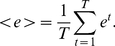
(8)Here 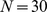 is the number of stocks and 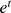 is the edge density for network 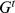 at time point 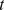 (for interval 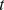) and 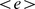 is the *mean edge density*, averaged over all 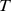 networks (intervals). The second measure we use is the *edges density difference*, 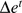, that can be found comparing network 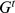 with 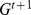 that means between two consecutive networks. This measure is given by
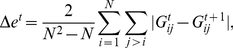
(9)

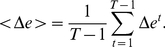
(10)Here 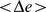 is the *mean edge density difference*, averaged over all consecutive networks. We want to mention that the definition of 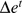 corresponds to the so called *graph edit distance* [29]–[31], which is a well known graph metric in quantitative graph analysis [32]. This branch of quantitative graph analysis is often referred to as *inexact graph matching* [30], [31] that addresses the problem of determining the structural similarity of graphs in an error-tolerant way [31], [32]. More precisely, the graph edit distance measure is based on applying graph transformations, i.e, a sequence of weighted graph edit operations that transform a given graph into another graph by producing minimal edit costs [32]. The third measure we use to quantify modification of the network structure is the number of nodes, 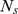, without connections to other nodes in the network. These nodes are isolated and separated from the rest of the system.

**Figure 1:**
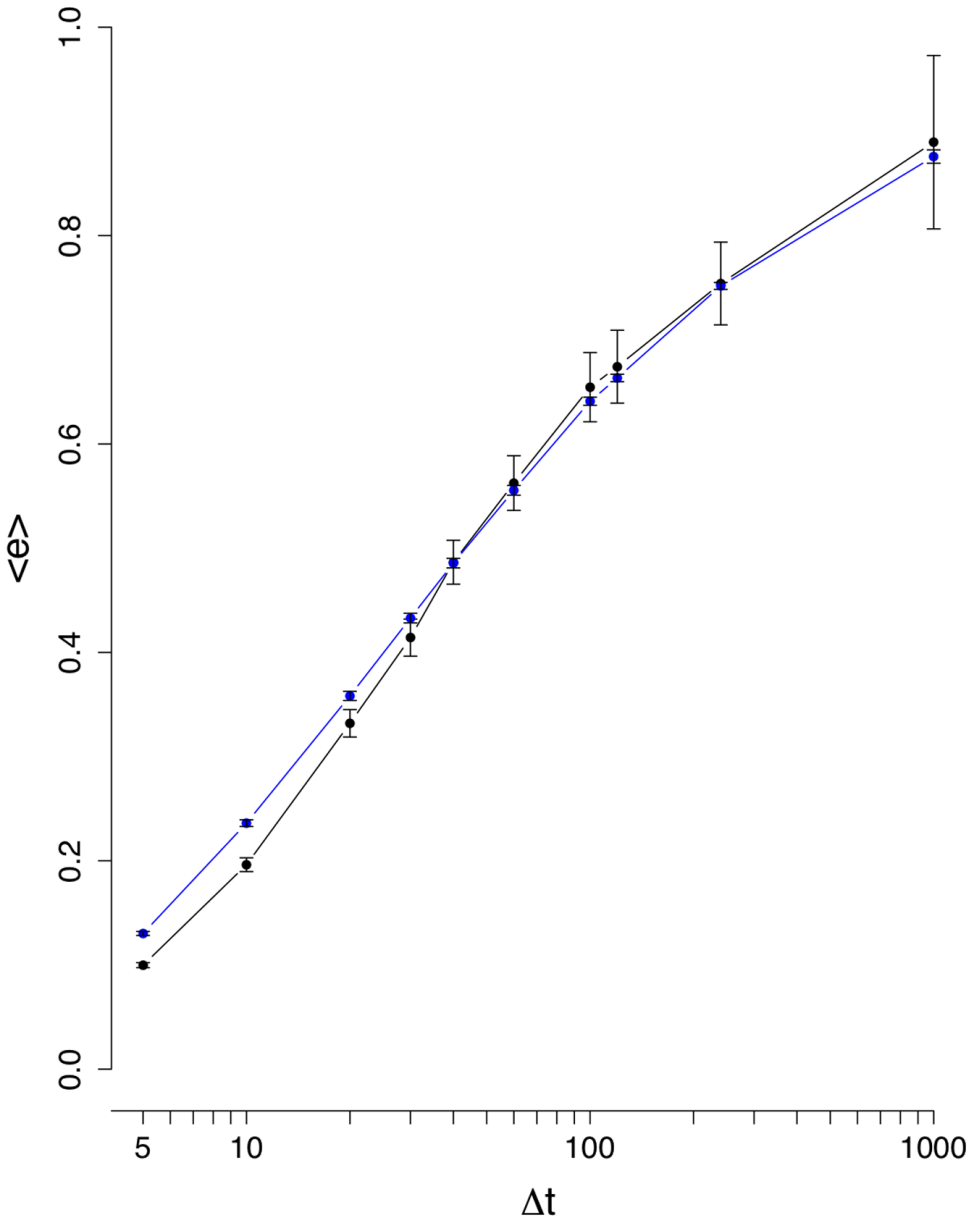
Mean edge density 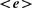 (black line). The blue curve corresponds to results from an inter-day randomization.

**Figure 2:**
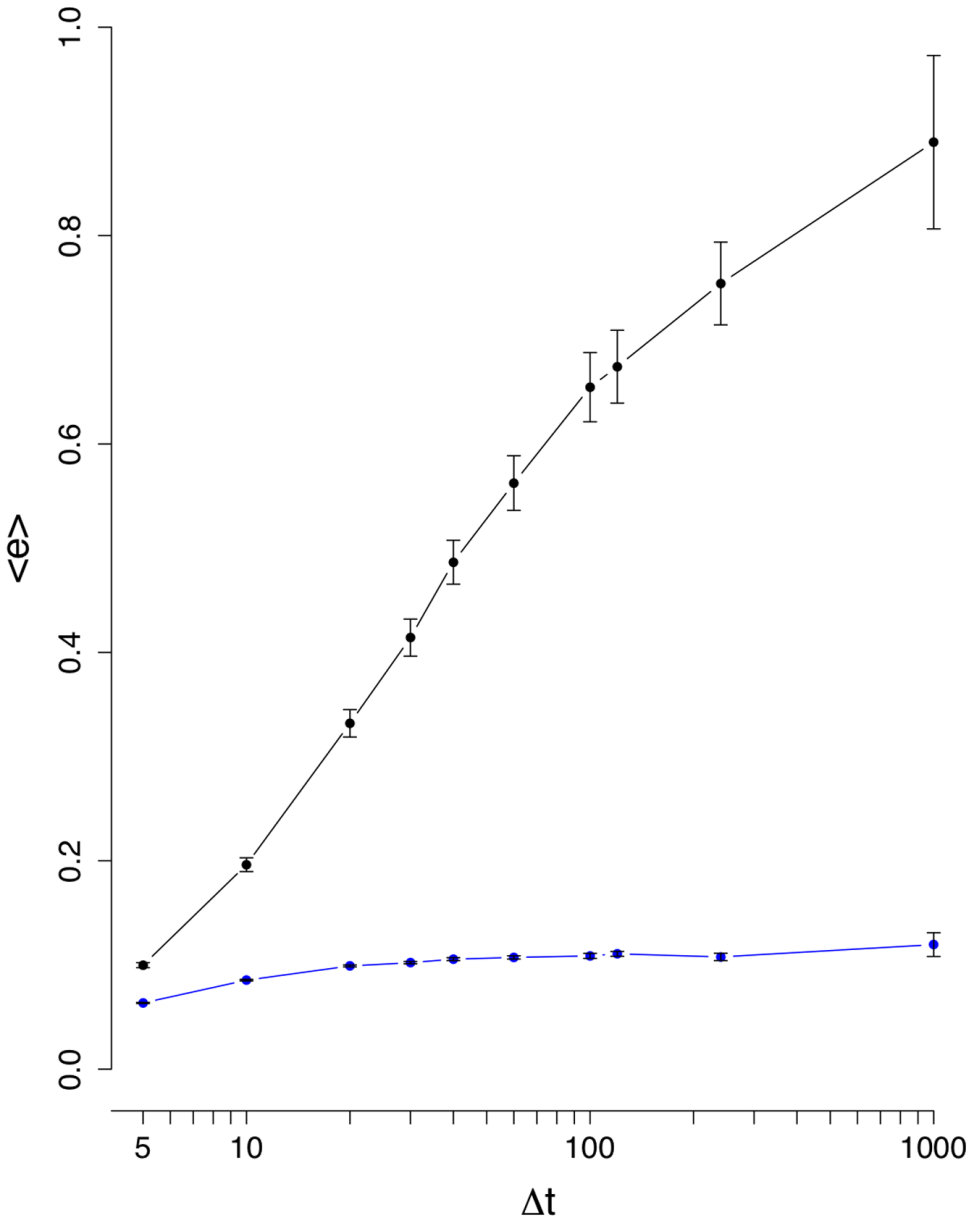
Mean edge density 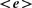 (black line). The blue curve corresponds to results from an intra-day randomization.

**Figure 3:**
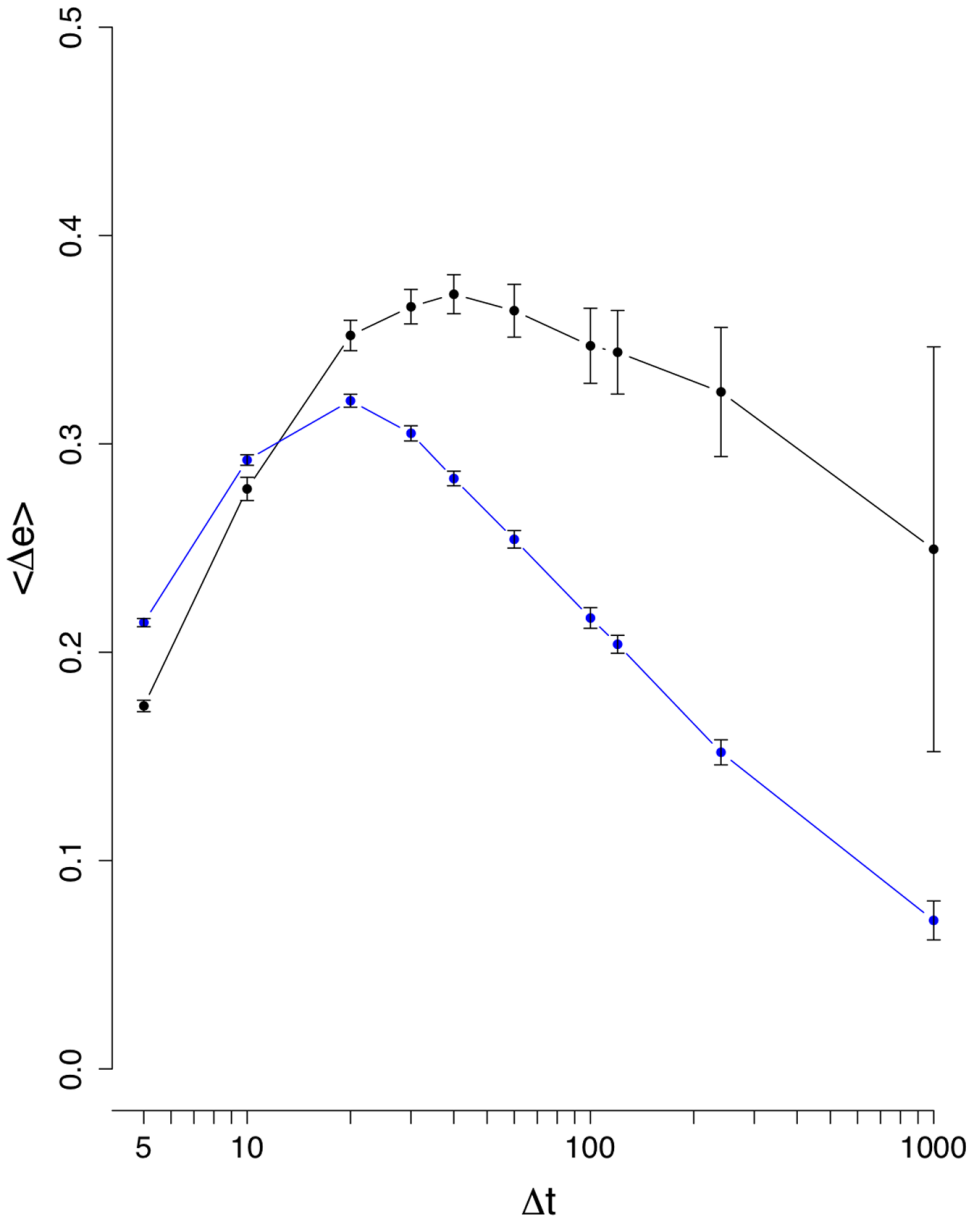
Mean edge density difference 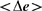 (black line). The blue curve corresponds to results from an inter-day randomization.

**Figure 4:**
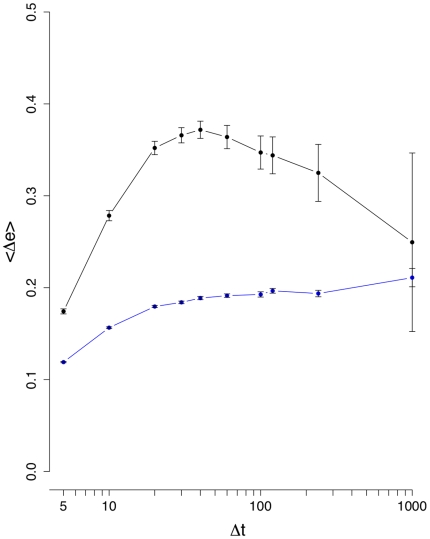
Mean edge density difference 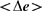 (black line). The blue curve corresponds to results from an intra-day randomization.

**Figure 5:**
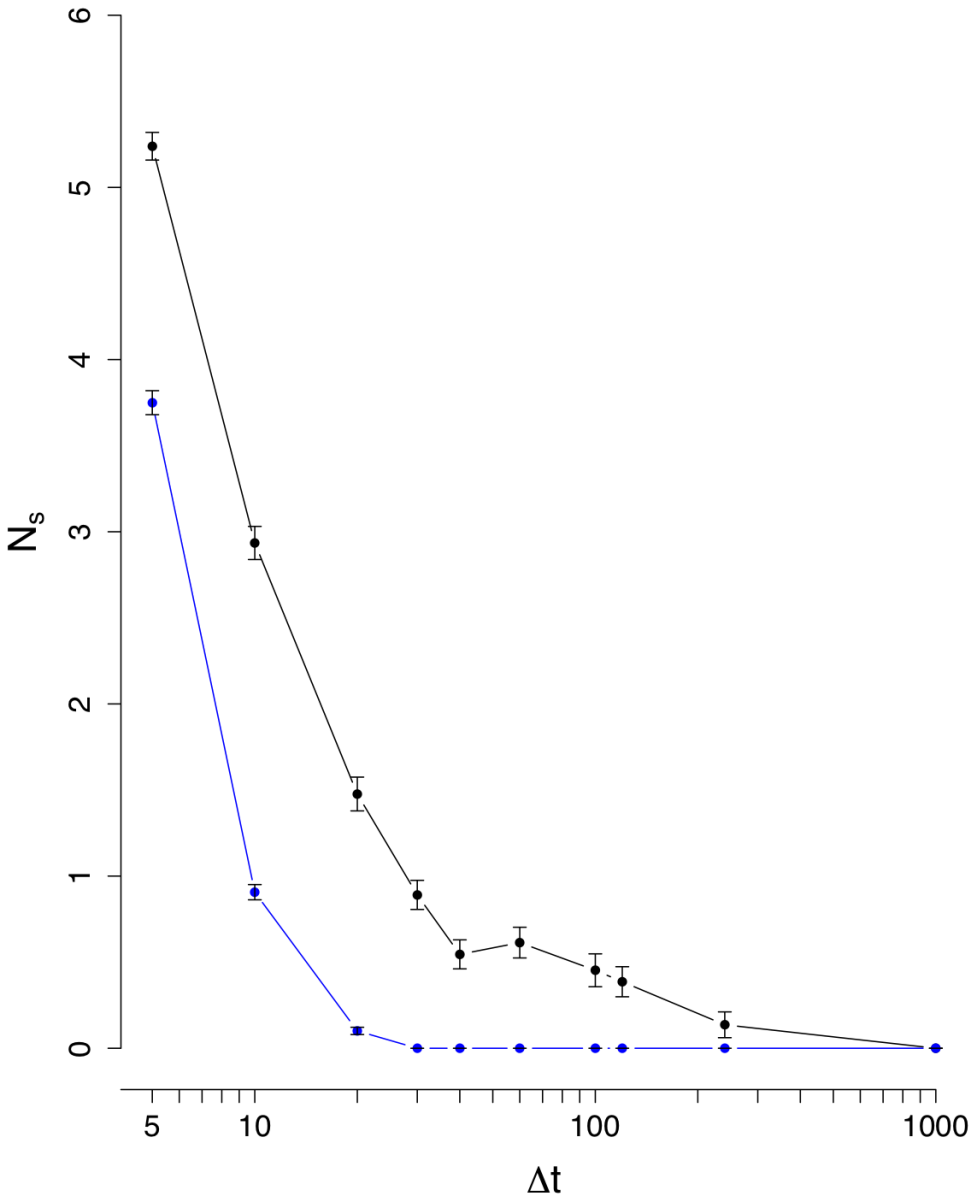
Mean number of unconnected nodes 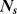 (black line). The blue curve corresponds to results from an inter-day randomization.

**Figure 6:**
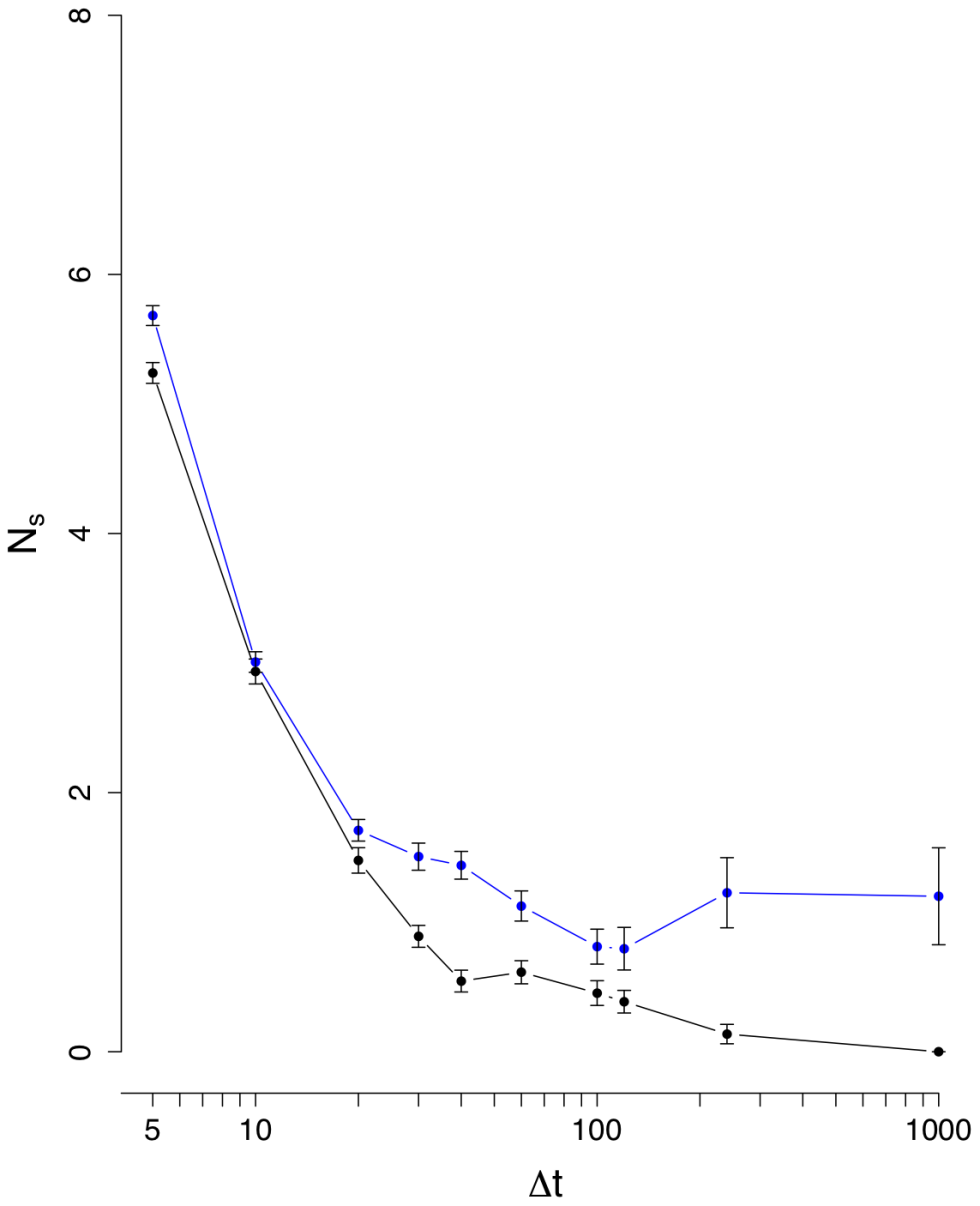
Mean number of unconnected nodes 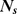 (black line). The blue curve corresponds to results from an intra-day randomization.

**Figure 7:**
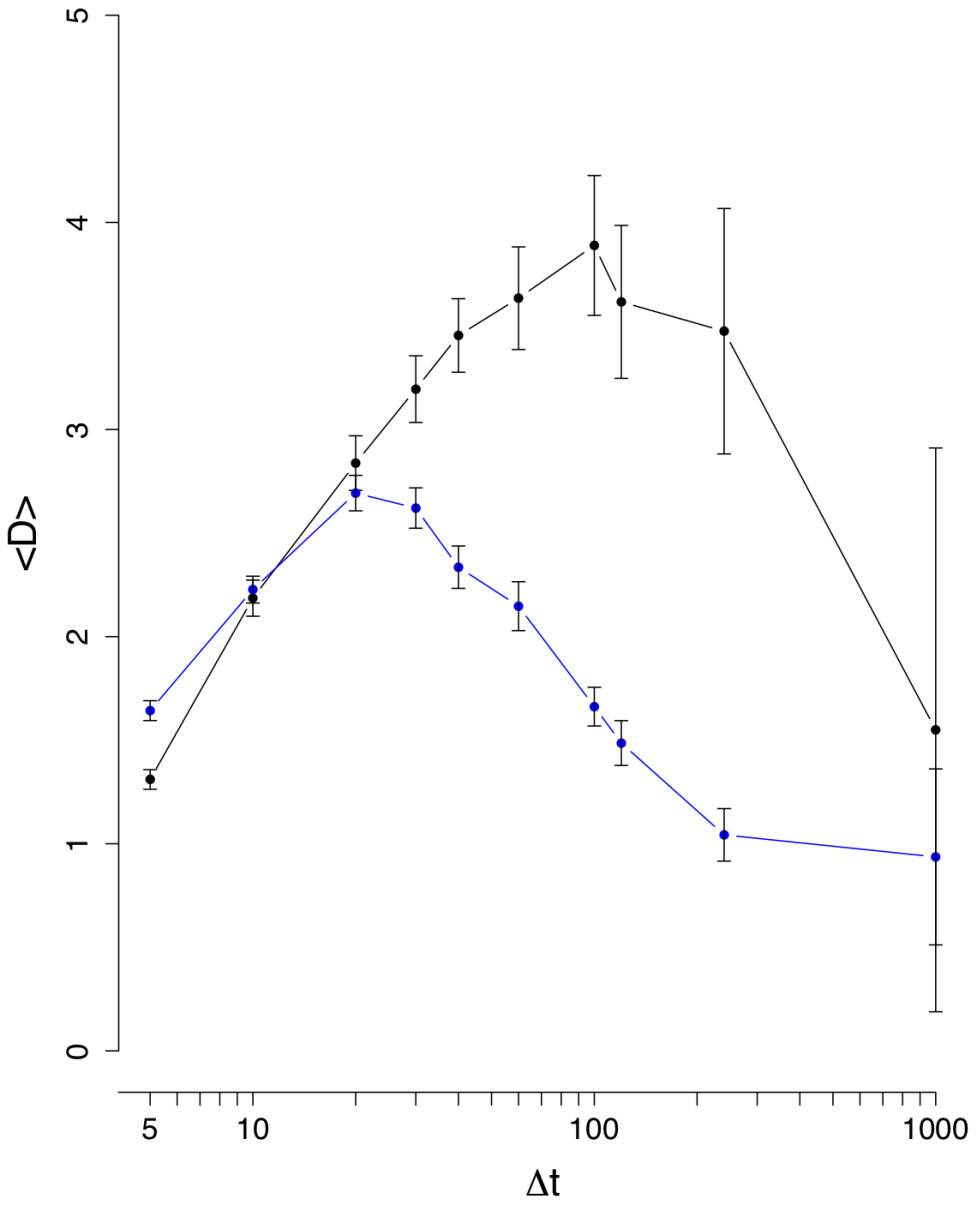
Mean Kullback-Leibler divergence 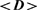 (black line). The blue curve corresponds to results from an inter-day randomization.

**Figure 8:**
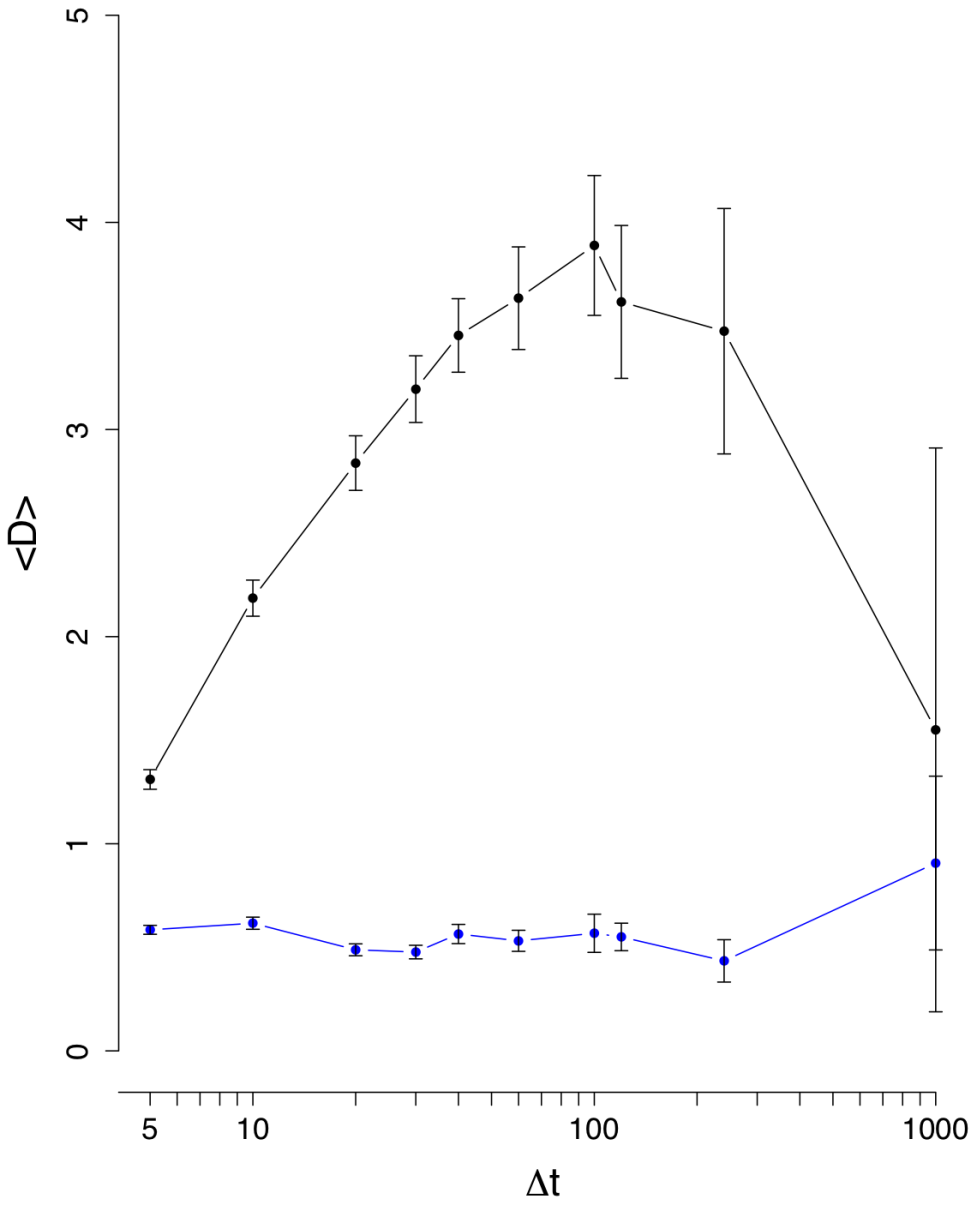
Mean Kullback-Leibler divergence 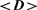 (black line). The blue curve corresponds form an intra-day randomization.

For reasons of comparison, we calculate not only 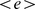, 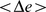 and 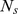 for the data of the stock market, but also for two randomized versions thereof. Randomized means, that we permute the data according to a scheme in order to destroy or establish new correlations randomly. We use two different randomization schemes. The first randomization scheme permutes the labels of the days 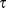 (not of the intervals) but conserves intra-day labels (stock labels). That means we change 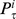 with 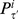 for all stocks 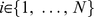 simultaneously. In the following, we call this kind of randomization *inter-day* randomization. The second randomization permutes in addition the stock labels. That means we change 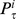 with 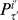 for each stock independently. For this reason we call it *intra-day* randomization. The figures 1, 3, 5 and 7 show the results for the inter-day randomization and the figures 2, 4, 6 and 8 the results for the intra-day randomization. For these figures, curves shown in blue correspond always to results from the randomizations.

Before we present our numerical results, we want to discuss the rationale for introducing the two randomization schemas. First of all, we would like to repeat that the major purpose in this paper is to identify a time scale that is most beneficial for the construction of financial networks. Here the definition of ‘beneficial’ is crucial in order to study this problem. There are at least two possibilities to find a definition, the first is an explicit definition the second an implicit one. An explicit definition would be most convenient, however, we are not aware of any higher principle that would allow a derivation thereof unequivocally. Hence, any explicit definition would be *ad hoc* and potentially difficult to defend. For this reason, we are pursuing the second path utilizing randomizations of the data. More precisely, a randomization of data can be interpreted as a removal of information from these data. Because we do not know how the networks should look like nor what structural properties they should have, we perform a comparative analysis. This comparative analysis compares networks constructed from (normal) data with networks constructed from randomized data allowing to detect differences or similarities. From this, we aim at identifying a time scale for which networks constructed from *normal* data are different to networks from randomized data, with respect to measures we are using in this paper. Because for such a time scale, the structural information of these networks is apparently different. The reason for using different randomizations is that, in general, more than one randomization is possible. In our case, we identified two randomizations that are sensible, given the specific context of our problem. Finally, we would like to emphasize that the fact that the randomized and normal networks appear similar, according to a measure employed, does not mean that this time scale may not be of any usage for the study of the stock market in general. Instead, it merely means that it does not appear to be advisable to use this time scale for the construction of financial networks for data of a similar type as the one used in this study.

For all following simulations, we obtain 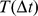 - depending on the time scale - different networks (see table 1). From these networks we estimate our four measures described above and their corresponding standard errors (shown as vertical bars in the figures). Fig. 1 shows the results for the mean edge density 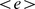. For this measure, the course of 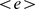 for the randomized data is very similar. Both curves start at low values for short time intervals 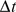, but increase steadily for larger intervals, until almost every stock is correlated with all other stocks. In this case, 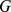 is almost fully connected. We would like to point out that the curves are similar but not identical because inter-day randomization permutes *trading days*, and not whole intervals. For the intra-day randomization, shown in Fig. 2, we observe a considerable difference for 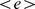. This demonstrates that the correlations among stocks within a day are stronger than the correlations between trading days. The intra-day randomization removes almost all correlations, which is the reason why 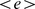 increases only slightly for larger values of 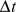. The interesting result from Fig. 1 is that for 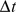 larger than about 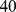 trading days both curves become indistinguishable, given the errors of the estimates. However, below this threshold they are different. Given the large sample sizes (see table 1) for these values, which is larger than 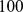, implies reliable estimates.

Fig. 3 shows the results for the mean edge density difference 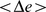. It is interesting to see that now a difference between the normal (black line) and inter-day randomized (blue line) data is clearly visible for all values of 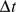, underlining the need to study several different measures, because each has its own sensitivity towards certain characteristics in the data. From Fig. 3 follows that between 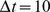 and 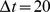, both curves separate strongly from each other and 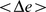 reaches its maximum for 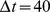 (normal data). For 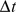 larger than 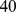 days, 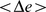 decays, because due to the longer interval sizes the correlations seem to reach an equilibrium resulting in consolidations of the correlations between stocks and, hence, a more stable structure of the financial networks. This means, if one wants to study the dynamical behavior of the stock market our results suggest to focus on 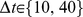 days. Using interval sizes below 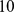 days seems not to be advisable because the noise seems to be of comparable strength as the signal. Again, the intra-day randomization shown in Fig. 4 is significantly different to the results for the normal data, and its low values can be explained due to the low number of significant correlations present in 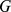 (see Fig. 2). The results for the mean number of unconnected nodes, 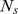, are shown in Fig. 5 and 6. Fig. 6 suggests also that below 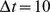 the signal and the noise are comparable in size, because the randomized and normal curves provide similar results and from Fig. 5 we see that 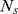 reaches an equilibrium for larger interval sizes.

Finally, we present results for one further measures, the mean Kullback-Leibler divergence. The mean Kullback-Leibler divergence [33], [34] for the degree distributions is given by
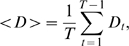
(11)with
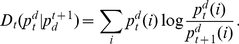
(12)The distributions 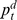 and 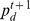 correspond to the degree distributions of the network at time (interval) 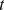 and 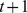 (interval). The Kullback-Leibler divergence evaluates the deviation of the degree distributions of consecutive networks and, hence, provides a measure to evaluate topological modifications regarding the connectivity of the networks. 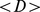 is a global measure because it compares distributions for the whole network, but in contrast to the edge density difference (9), which is also a global measure, 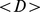 does not assess modifications of individual vertices but of the whole collective. For this reason 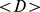 is less sensitive against individual degree modifications as long as the whole network maintains a certain distribution. That means 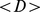 does not detect individual degree modifications and, hence, is more abstract then (9).

Fig. 7 and 8 show our results for the mean Kullback-Leibler divergence. The qualitative results for the mean Kullback-Leibler divergence are similar to the results for the edge density difference (Fig. 3 and 4). For 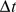 larger than 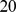, the normal and inter-day randomized curves separate from each other indicating that successive networks are more and more dissimilar, because the degree distributions become more and more dissimilar. For larger 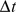 values, both curves come closer together (this holds also for the intra-day randomization shown in Fig. 8) indicating an decreasing amount of signal in the networks respectively their degree distributions. A difference between 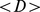 and 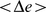 is the location of the maxima, which is at 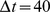 for 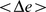 and 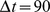 for 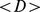.

## Discussion

In this paper we investigated the construction of financial networks. We used the correlation matrix to define undirected and unweighted networks, resulting from the application of statistical hypothesis tests to the correlation coefficients. This results in statistically significant networks, wherein stocks correspond to nodes and edges correspond to non-vanishing correlation coefficients [7]. The novel contribution of this paper is a systematic investigation of the influence, the length of the used time interval, 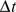, has on the resulting networks respectively on their structure. The goal was to find a time scale, 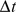, that results in the most meaningful networks. Here we consider a network structure to be meaningful, if it is a reflection of the *current* state of the stock market. Our underlying hypothesis is based on the assumption that the stock market is a dynamical system [35]. For this reason it appears reasonable not to represent the market by just one network for the whole time series of all trading days, but to construct many networks, each for a much shorter time interval. This way the networks may capture and, hence, represent characteristic information of the corresponding time intervals reflecting the state of the dynamical system. Intuitively, it seems to be clear that networks that have many unconnected nodes (low 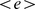 and high 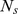 values) and networks that are almost fully connected in average (high 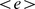 values), are not meaningful. From an economics point of view, it is very difficult to provide an exact definition of network properties that should be present because our knowledge of the working mechanism of the stock market is far from being complete. For this reason, we studied the behavior of four measures (three graph-theoretical and one information-theoretical measure) in dependence on 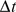 comparatively by means of randomized data. Our results indicate that a time scale from more than 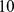 to 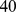 trading days, corresponding to an interval between 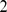 weeks and 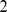 months, seems to be most favorable for the construction of the financial networks. Using a time scale that is shorter or much longer, results in networks that are either very sparsely connected with a high number, 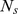, of unconnected nodes (see Fig. 5 and Fig. 6) or in networks that are almost fully connected (see Fig. 1 and 2). Clearly, the utility of the networks depends strongly on the scientific question under consideration, however, a different time scale seems not to be advisable, because otherwise the properties of the networks are *in average* quite extremal and not just for some of the networks.

Regarding potential applications, our results clearly indicate that networks should be constructed from more than 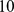 trading days in order to reduce the amount of noise in the constructed networks. Again, this recommendation holds only for financial data of the same type as ours (daily closing prices). This implies that the lower bound found by our analysis is the more relevant one for applications in time series analysis, because 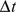 defines the *resolution* in the application of the financial networks.

From a more abstract point of view the topic of our paper is complementary to studies investigating the generation of (complex) networks [36]–[39]. Such studies define usually a stochastic procedure consisting of basic rules, whose iterative application generates a network with certain structural properties. In contrast, we obtained networks not by a generation but a construction (estimation) procedure which is based on data. It would be interesting to study structural properties of financial networks constructed by our procedure and to compare them with known network models in the literature. It should not surprise to find similarities because also many recent network models that give rise to power law distributions in the degrees form special cases of classic models by Yule or Simon [40], [41] (see [37] for a thorough discussion). For this reason such a comparison might give further insights into the dynamical processes of financial networks and their constituting stocks/companies.

We consider our results as an important step that hopefully facilitates the interest and the usage of networks in the context of financial markets, because to be able to analyze financial networks, first, we need to define them. As we pointed out above, the time scale used to construct the networks plays a crucial role in this endeavor. In our future work, we will investigate networks that are constructed by using the relevant time scale found in this article. We are of the opinion that network-based analysis methods will gain even more attention in the analysis of financial markets in the near future, because the analysis of networks implies the analysis on a systems level [42].
